# College students’ influence on COVID-19 vaccination uptake among seniors in China: a protocol of combined cross-sectional and experimental study

**DOI:** 10.1186/s12889-023-16209-2

**Published:** 2023-07-10

**Authors:** Junye Bian, Zhihui Guo, Weijie Zhang, Xinyi Li, Caijun Sun, Xuelian Xu, Huachun Zou

**Affiliations:** 1 School of Public Health (Shenzhen), Sun Yat-sen University, Shenzhen, China; 2 School of Literature and Education, Bengbu University, Bengbu, China; 3 School of Public Health, Fudan University, Shanghai, China; 4 School of Public Health, Southwest Medical University, Luzhou, China; 5 Kirby Institute, University of New South Wales, Sydney, Australia

**Keywords:** COVID-19, College students, Older adults, Vaccination, China

## Abstract

**Background:**

College students generally have good knowledge about COVID-19 and may facilitate COVID-19 vaccination in family. The purpose of this study is to understand college students’ willingness to persuade their grandparents to initiate COVID-19 vaccination and the effect of their persuasion.

**Methods:**

A combined cross-sectional and experimental study will be conducted online. In the cross-sectional study (Phase I), eligible participants are college students who are aged ≥ 16 years and have at least one living grandparent aged ≥ 60 years who has/have not completed the COVID-19 vaccination. Participants self-complete Questionnaire A to collect information on the socio-demographics of themselves and their grandparents, their knowledge about older adults’ COVID-19 vaccination, as well as Health Belief Model (HBM) and Theory of Planned Behavior (TPB) predictor variables. The primary outcome at Phase I is college students’ willingness to persuade grandparents to receive COVID-19 vaccines. Those who are willing to persuade grandparents and participate in a follow-up survey will be invited to participate in a randomized controlled trial (Phase II). At Phase II, eligible participants are those who have at least one living grandparent aged ≥ 60 years who completed the COVID-19 initial vaccination series but has/have not received a booster dose. At the baseline, participants self-complete Questionnaire B to collect information on individual grandparents’ COVID-19 vaccination status, attitude towards and intention to COVID-19 booster dose. Participants will then be randomly allocated 1:1 to either intervention arm (one-week smartphone-based health education on older adults’ COVID-19 vaccination plus two weeks’ waiting period) or control arm (three weeks’ waiting period). At the end of week three, participants in both arms self-complete Questionnaire C to collect information on their grandparents’ COVID-19 vaccination status. The primary outcome at Phase II is the uptake rate of COVID-19 booster dose among grandparents. Secondary outcomes include grandparents’ attitude and intention to get a COVID-19 booster dose.

**Discussion:**

No previous study had measured the effect of college students’ persuasion on COVID-19 vaccination uptake in older adults. Findings from this study will provide evidence for innovative and potentially feasible interventions that further promote COVID-19 vaccination in older adults.

**Trial registration:**

Chinese Clinical Trial Registry: ChiCTR2200063240. Registered 2 September 2022.

**Supplementary Information:**

The online version contains supplementary material available at 10.1186/s12889-023-16209-2.

## Background

Coronavirus disease 2019 (COVID-19), caused by the SARS-CoV-2 virus, has caused hundreds of millions of infections and millions of deaths worldwide [1, 2]. Among the whole population, older adults tend to be more prone to serious infections, medical complications and deaths after infection with COVID-19 because of underlying diseases and compromised immunity [3, 4]. In China older adults aged 60 years and older accounted for 31% and 81% of the total infected and death cases of COVID-19 [5]. China is an aging society. The population of people aged 65 years and older was just nearly 165 million in 2019, which brings huge challenges to the prevention and control of COVID-19 in China [6]. Hence, older adults are listed as key groups in China’s ninth edition (the most up-to-date version) of COVID-19 prevention and control plan [7].

COVID-19 vaccination is proven to be effective and safe in preventing deaths and other severe consequences caused by COVID-19 among older adults [8]. Besides, booster vaccination is also essential because of the decline in the titers of antibodies over time after vaccination [9, 10]. With the continuous development of vaccines, countries around the world have successively implemented phased vaccination programs [11, 12]. China implemented a nationwide free vaccination program in January 2021 and started a booster dose in September 2021 [13]. As of July 7, 2022, the number of people aged 60 years and older who have been vaccinated and fully vaccinated, accounted for 89% and 84% of older adults respectively, and more than 170 million older adults have received a booster dose [14]. The rollout of COVID-19 vaccines for older adults in China is generally progressing well, but there are still some problems. First, the coverage of COVID-19 booster vaccination for older adults is suboptimal and reaches a plateau [15]. Second, the vaccination coverage of older adults aged 80 years and older need to be improved [14]. Hence, the promotion of vaccination among older adults needs to be further strengthened.

Various measures have been adopted to promote COVID-19 vaccination uptake. For example, a trial in Sweden found that guaranteed cash incentives increased COVID-19 vaccination uptake by 4% [16]. Some other countries provided non-cash rewards, such as free rice/eggs in mainland China, hummus in Israel, and blenders in India, but the impact of these approaches on vaccination remains largely unassessed [17]. Up to now, there are few cost-effective ways to promote vaccination in older adults. Compared to previous ways of promotion based on money or commodity, a randomized controlled trial in Japan based on evolutionary theory found that an information intervention targeting the fundamental motive of kin care can significantly increase willingness to receive COVID-19 vaccination [18]. Furthermore, most older adults in China preferred to get healthcare information from interpersonal sources, [19] and a survey of vaccination intentions among older adults indicated that recommendations from family members may prompt older adults to agree to vaccinate against COVID-19 [20]. These studies show the importance and potential feasibility of family members in promoting vaccines.

As a family member with good knowledge, attitude and practice (KAP) towards COVID-19, college students may be able to receive and then transfer public health messaging to their older family members [21]. Besides, under the influence of traditional Chinese filial piety culture and modern post-figurative culture, college students usually have a good connection with older adults in their family. Additionally, interventions developed based on intergenerational bond between grandparents and grandchildren have also proven effective in alleviating mental problems of older adults [22]. These all indicate that college students may be a natural group to influence older adults’ health decision making. However, college students’ influence on older adults’ decision making of COVID-19 vaccination, has not been studied.

Our study comprises a combination of a cross-sectional study and a subsequent randomized controlled trial. The cross-sectional study aims to assess college students’ willingness to persuade their grandparents to initiate COVID-19 vaccination and investigate factors associated with their willingness. The randomized controlled trial aims to evaluate the impact of college students’ persuasion on grandparents’ COVID-19 booster dose vaccination.

## Methods and analysis

### Study design and setting

A combined cross-sectional and experimental study will be conducted online in China to understand college students’ willingness to persuade their grandparents to initiate COVID-19 vaccination and the effect of their persuasion.

**Fig. 1:**
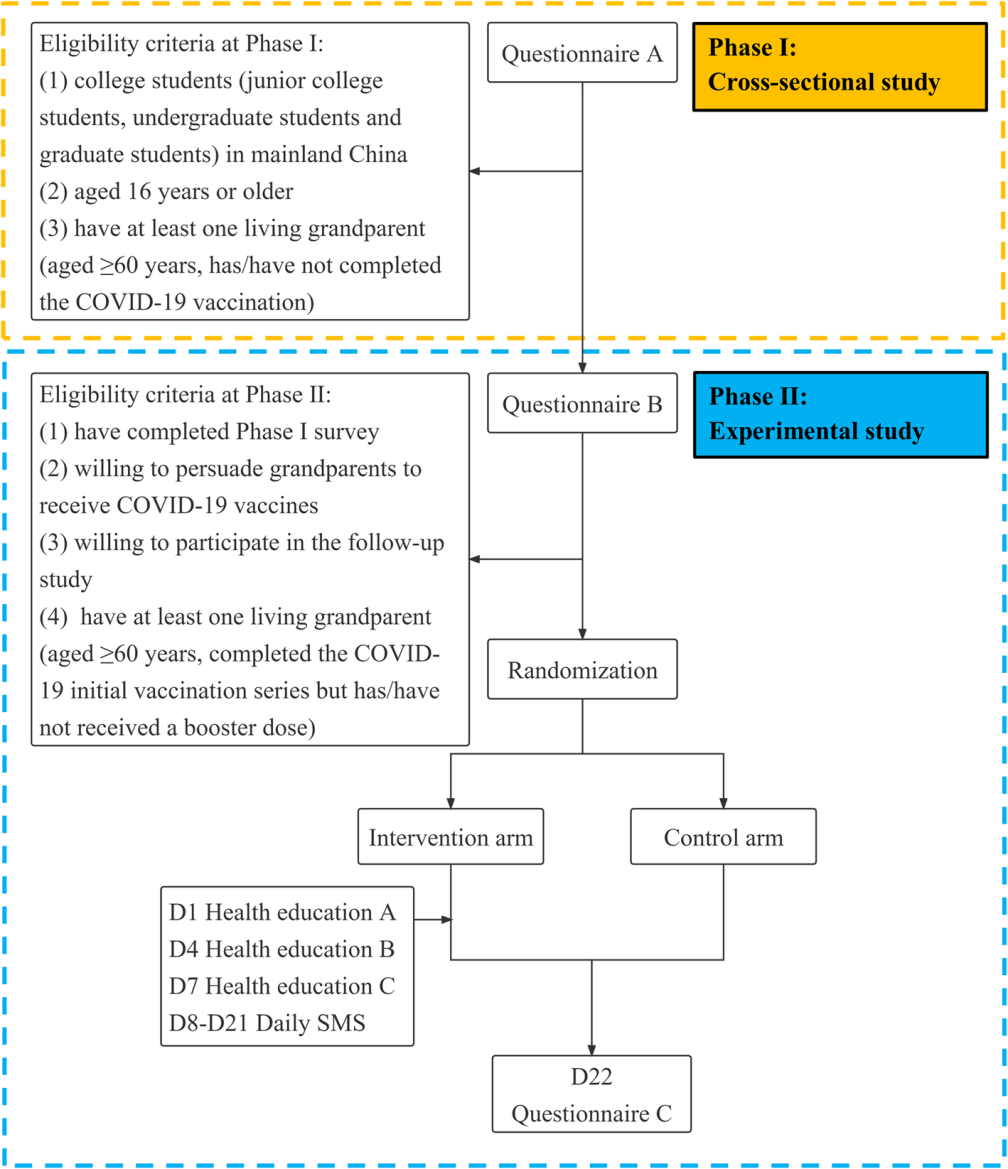
Flow chart of the study

The study has two study phases (Fig. 1; Table 1), including a cross-sectional study (Phase I) and a randomized controlled trial (Phase II). Electronic informed consent will be obtained at the beginning of both phases.

At Phase I, college students will be recruited by convenience sampling. Our online survey will be distributed by college students via the social network including WeChat (WeChat is a social media app developed by Tencent, Shenzhen, China). College students will be asked about their willingness to persuade their grandparents to initiate COVID-19 vaccination. Those willing to persuade and participate in a follow-up survey will be invited to participate in the next phase.

At Phase II, considering the representativeness of the sample, stratified sampling between four regions of China (East, Central, West and Northeast) was conducted when recruiting participants. At this phase, eligible participants will be randomly allocated 1:1 to either intervention arm (one-week smartphone-based health education on COVID-19 vaccination in older adults plus two weeks’ waiting period) or control arm (three weeks’ waiting period). At the end of week three, the actual uptake of grandparents’ COVID-19 vaccination will be obtained via college students.

**Table 1:** Schedule of the study events

	Cross-sectional study (Phase I)	Experimental study (Phase II)					
EVENTS	Enrollment	Eligibility reconfirmation	Follow-up				Close-out
TIMEPOINT	Month − 1	0	*Day 1*	*Day 4*	*Day 7*	*Day 8–21*	*Day 22*
ENROLLMENT:							
Eligibility screening	X	X					
Informed consent	X	X					
Allocation		X					
INTERVENTIONS:							
Web-based education package			X	X	X		
Manipulation checks			X	X	X		
Daily short message service (SMS) reminder						X	
ASSESSMENTS:							
Socio-demographics of students and grandparents	X						
Grandparents’ COVID-19 vaccination status	X	X					X
Grandparents’ attitude towards COVID-19 booster dose		X					X
Grandparents’ intention to get COVID-19 booster dose		X					X

### Inclusion criteria

Individuals who meet the following criteria are eligible at Phase I: (1) college students (junior college students, undergraduate students, graduate students) in mainland China, (2) aged 16 years or older, (3) have at least one living grandparent aged ≥ 60 years who has/have not completed the COVID-19 vaccination recommended by Chinese health authority (including not vaccinated, not completed the initial vaccination series or completed the initial vaccination series but has/have not vaccinated a booster dose).

Those who meet the following criteria are eligible at Phase II: (1) have completed Phase I survey, (2) have the willingness to persuade grandparents to receive COVID-19 vaccines, (3) willing to participate in the follow-up study, (4) have at least one living grandparent aged ≥ 60 years who completed the COVID-19 initial vaccination series but has/have not received a booster dose. Considering that the vaccination status of older adults may change during the time interval between the two phases, eligibility of Phase II will be double-checked by the baseline questionnaire at Phase II (Questionnaire B).

### Sample size

At Phase I, we use the formula $N=\frac{{Z}_{\text{1-}\alpha }^{2}\times p\times (1-p)}{{\delta }^{2}}$ to calculate the sample size. Given an alpha value of 0.05 and the permissible error δ of 0.02, assuming the proportion of college students willing to recommend vaccination for older adults at home as 50%, a minimum of 2401 subjects are required for this quantitative survey.

At Phase II, we use the formula $N=\frac{[{Z}_{1-\alpha /2}\times \sqrt{\bar{p}\times \bar{q}\times (1+\frac{1}{k})}+{Z}_{1-\beta }\times \sqrt{{p}_{1}\times {q}_{1}+\frac{{p}_{2}\times {q}_{2}}{k}}{]}^{2}}{({p}_{1}-{p}_{2}{)}^{2}}$ to calculate the sample size. Using parameter, the rate of booster vaccination in older adults is 2.5%, [23, 24] and this figure is expected to increase to 4 times after information intervention, [25] which requires a sample size of 162 eligible older adults in each group to achieve 80% power at a 2-sided 5% level of significance to detect a net difference. Through the preliminary data in Phase I, at least 101 eligible college students are needed to provide the required sample size of eligible older adults.

### Data collection

Three questionnaires will be set up via “Wenjuanxing”, a Chinese online platform providing survey functions. These questionnaires were evaluated by a panel of experts and were revised based on their suggestions and a pre-survey conducted among 30 college students. The protocol including the questionnaires was revised based on information collected in the pre-survey. Table 2 depicts informative to be collected in the study.

At Phase I, Questionnaire A will be used to collect socio-demographics and background information of both college students and their grandparents. Purpose-designed questions have been developed to examine college students’ knowledge about COVID-19 vaccination in older adults. Besides, the Health Belief Model (HBM) and the Theory of Planned Behavior (TPB) model, both of which have been widely used in the context of vaccination, are also included in Questionnaire A to explain and predict college students’ willingness to persuade grandparents to receive the COVID-19 vaccine [26, 27].

At Phase II, Questionnaire B and Questionnaire C will be respectively used to collect information on COVID-19 vaccination status, attitude towards and intention to COVID-19 booster dose of participants’ grandparents at the beginning and end of this phase.

In three questionnaires, we provide clear options for objective questions. Several quality-control questions will be included in our study questionnaires, and the consistency of answers will be examined by trained investigators. All investigators have completed standard operating procedure training and data management training. For subjective questions, traditional five-point Likert’s scales are used to assess college students’ knowledge about COVID-19 and COVID-19 vaccination in older adults, HBM, TPB, grandparents’ attitude towards and intention to get COVID-19 booster dose (Additional files 1 & 2). Of note, in order to ensure the reliability and validity, all the Likert’s scales mentioned above are adapted from previous studies [18, 28].

**Table 2:** Summary of information to be collected

Population	Modules	Questionnaire code(s)
College students	Socio-demographics	A
	Knowledge about COVID-19 vaccination in older adults	
	HBM predictors for willingness to persuade grandparents to get COVID-19 vaccination	
	TPB predictors for willingness to persuade grandparents to get COVID-19 vaccination	
	Willingness to persuade grandparents to get COVID-19 vaccination	
	Willingness to participate in Phase II	
Grandparents	Socio-demographics	A
	COVID-19 vaccination status	A&B&C
	Attitude towards COVID-19 booster dose	B&C
	Intention to get COVID-19 booster dose	B&C

A randomized controlled trial will be conducted at Phase II. Questionnaire A will be assigned to the college students at Phase I. Questionnaire B and Questionnaire C will be used to collect grandparents’ information at the beginning and end of Phase II

### Cross-sectional study

Data of the cross-sectional study will be collected via Questionnaire A. Participants will receive a symbolic reward (CNY 1, or USD 0.15) after completing the questionnaire. At this phase, the dependent variable is defined as college students’ willingness to persuade grandparents to receive COVID-19 vaccines. The independent variables are grouped into five blocks:

1. College students’ socio-demographics: sex, age, ethnic, educational background (junior college students, undergraduate students and graduate students), major and monthly household income.
2. College students’ knowledge of COVID-19 vaccination in older adults included 7 items on a 5-point scale (from “1 = strongly disagree” to “5 = strongly agree”). The variable will be transformed to categorical (No, Yes), with a cut-off value determined by restricted cubic spline.
3. HBM predictor variables for college students’ willingness to persuade grandparents to get COVID-19 vaccination: perceived susceptibility (included 2 items), perceived severity (included 3 items), perceived benefits (included 2 items), perceived barriers (included 1 items) and cues to action (included 3 items). Items in the HBM will be measured on a 1–5 scale (from “1 = strongly disagree” to “5 = strongly agree”). The items of perceived severity and cues to action will be reverse-scored.
4. TPB predictor variables for college students’ willingness to persuade grandparents to get COVID-19 vaccination: attitude (included 1 items), subjective norms (included 3 items) and Perceived Behavioral Control (included 1 item). Items in the TPB will be measured on a 1–5 scale (from “1 = strongly disagree” to “5 = strongly agree”).
5. Grandparents’ socio-demographics: sex, age, educational background, type of residence (urban or rural), cohabitation history (living together with grandchildren for one year or more), living conditions (living alone, only with spouse, with other family members, living in pension institutions), outdoor activities (measured by frequency), chronic disease (having one or more of the following: cardiovascular diseases, diabetes mellitus, tumor, chronic respiratory diseases, liver cirrhosis, chronic renal failure, nervous system diseases and mental disease).

In addition, college students’ willingness to participate in Phase II and grandparents’ actual COVID-19 vaccination status after persuasion will also be included in Questionnaire A to select eligible participants for the next phase.

### Randomized controlled trial

Eligibility for Phase II will be judged by Questionnaire A and confirmed by Questionnaire B because COVID-19 vaccination status may change during the interval between the two phases. Eligible participants will be assigned to the intervention or control arm at a 1:1 ratio chronologically, according to the sequentially numbered allocation list, which was determined by a series of random numbers generated with Microsoft Excel 2010 (Microsoft Corp., Redmond, WA, USA) prior to recruitment. College students with the order of enrollment corresponding to the smaller half of random numbers will be allocated to the intervention arm, and the rest will be allocated to the control arm. This experimental study is single-blind whereby participants will be unaware of what interventional message will be given to them. Of note, to ensure the reliability of the data, all the participants will receive a simple investigate training in word and be asked to contact their grandparents before completing the Questionnaire B.

At Phase II, a web-based health education package about COVID-19 will be provided to college students in the intervention arm for one week via the research’s WeChat Official Account (a China-based marketing platform with the functions of gathering followers and sending them targeted content). The intervention arm will receive a 5-min web-oriented multicomponent education package at Day 1, 4 and 7, whereas the control arm will not receive this package, reflecting the situation of older adults receiving COVID-19 booster dose under the influence of the current vaccine promotion policy (Table 3). The intervention package consists of two parts: COVID-19 related information and testimony from older adults who received COVID-19 booster.

Related information will be established based on knowledge, attitude, and practice (KAP) model, a framework which has been used to develop behavior change interventions in other settings, and be presented in the form of questions and answers and cover the accompanying themes: (1) current situation and relevant policies of COVID-19 vaccination among older adults in China, (2) reasons to get COVID-19 booster doses, (3) benefits and effectiveness of COVID-19 booster, (4) safety of COVID-19 vaccines, (5) indications and contraindications of vaccination, (6) precautions for vaccination [29]. These materials could be expected to specifically address these barriers against COVID-19 vaccination among older adults, including concerns about vaccine effectiveness, adverse effects, and being unsuitable for vaccination [30]. Additionally, some research found that narrative persuasion is also an effective means to promote vaccination [31, 32]. Based on the cases of older adults who successfully received COVID-19 vaccine reported by mainstream media, appropriate modifications are made to form intervention materials for narrative persuasion.

After the 7-day intervention, two weeks will be left for the participants in the intervention arm to persuade their grandparents to receive booster injections. Meantime, they will receive a daily short message service (SMS) reminder written on the basis of loss frames, which turned out to have a stronger persuasive effect than gain frames, to remind them to persuade their grandparents [32, 33]. At Day 22, participants in both arms will complete Questionnaire C to record their grandparents’ latest vaccination status and changes on their attitude and willingness towards booster doses.

To improve follow-up rates, participants will receive another symbolic reward (CNY 3, or USD 0.45) for each completed questionnaire. If they complete all follow-ups, they will receive an additional reward (CNY 8, or USD 1.2).

**Table 3:** Schematic diagram of intervention

Content	Day 1	Day 4	Day 7	Day 8–21
Current situation and relevant policies of COVID-19 vaccination among older adults in China	√			
Reasons for receiving a COVID-19 booster dose	√			
Benefits and effectiveness of COVID-19 booster	√			
Safety of COVID-19 vaccines	√			
Indications and contraindications of COVID-19 vaccination		√		
Precautions for COVID-19 vaccination		√		
Testimony from older adults who received COVID-19 booster			√	
Daily SMS reminder^a﻿^				√

^a^SMS refers to short message service

### Manipulation checks

At the end of each intervention material, two simple questions about the content will be left to check manipulation. Only by answering both of the questions correctly can participants in the intervention arm get an additional symbolic reward (CNY 1, or USD 0.15).

### Outcomes

At Phase I, the primary outcome is college students’ willingness to persuade grandparents to receive COVID-19 vaccines, measured by the item “Would you like to encourage COVID-19 vaccination for your grandfather/grandmother?” (No, or Yes). In this item, college students were asked to report their intention to encourage COVID-19 vaccination for each of their grandparents. At Phase II, the primary outcome is grandparents’ uptake rate of COVID-19 booster dose, measured by the item “Have you (grandparents) received a COVID-19 booster dose?” (No, or Yes). Secondary outcomes include grandparents’ scores of attitude and intention to get a COVID-19 booster dose.

### Statistical analysis

All analysis will be performed using R (V.3.6.1, Foundation for Statistical Computing, Vienna, Austria). All effects will be estimated with 95% CI and *P* values. Statistical significance will be taken as two-sided *P* values < 0.05.

According to the types of the variables, descriptive statistical analysis will be carried out for all variables and outcomes, including count, frequency, ratio, mean, standard deviation, and so on. To test the reliability of all the Likert’s scale, Cronbach’s α test will be used.

At Phase I, the primary outcome is college students’ willingness to persuade their grandparents to receive COVID-19 vaccine. Considering that older adults will be nested in families, the condition that all participants are independent, as required for general logistic regression, does not hold. Thus, the multilevel logistic regression model will be necessary. Assuming that each college student has a different intercept, we will apply random intercept, fixed slop models to investigate factors associated with college students’ willingness. The fixed effects will be reported as adjusted odds ratio (AOR) while random effects will be reported by variance, intraclass correlation (ICC). The Akaike’s information criterion (AIC) will be used to determine the goodness of fit.

At Phase II, Chi-squared test will be used for between-group comparison of grandparents’ uptake of COVID-19 booster dose. Incidence rate ratio (IRR) and 95% CIs will be calculated with Poisson regression, adjusted for grandparents’ sex, age, type of residence, educational background, cohabitation history, living conditions, outdoors activities and chronic diseases. Subgroup analyses will be conducted according to grandparents’ sex, age, type of residence, educational background, cohabitation history and chronic diseases. We will use ANCOVA to analyze grandparents’ score of attitude towards COVID-19 booster dose and intention to get a COVID-19 booster dose. Outcomes will be assessed using both intention-to-treat (ITT) and per-protocol (PP) sets. The ITT analysis will calculate outcomes for all enrolled grandparents. The PP analysis will only include grandparents of grandchildren who successfully completed the questions embedded within the health education materials and completed a follow-up survey. To account for loss-to-follow-up, we will use multiple imputation by chained equations to impute missing values.

### Ethics and dissemination

The study has been approved by the ethical review committees at School of Public Health (Shenzhen), Sun Yat-sen University. Participants’ identifiable information (name, ID card number, address, etc.) will not be collected. Meanwhile, for ethical reasons, all participants in the control arm will receive the same intervention materials as the intervention arm at the end of the study.

## Discussion

As college students play an increasingly important role in family decision-making, grandchild-mediated vaccine promotion strategy at the family level shows potential value. This study protocol focuses on this interesting issue and is the first to describe an approach combing a cross-sectional and a randomized controlled trial to discuss the effect of college students’ persuasion on COVID-19 vaccination uptake in older adults. First, a cross-sectional study will be applied to help us understand the willingness of college students’ persuasion and related factors, which indicate the preliminary feasibility of a latent and cost-effective new strategy to promote vaccination in older adults. The reason behind those college students who are reluctant to persuade may inform future interventions aimed at improving their willingness to persuade. Meanwhile, cross-sectional study in our protocol plays a dual role in providing theoretical assumptions and participant recruitment for randomized controlled trial, which is more efficient than two separate studies otherwise. Second, combined with traditional socio-demographics, HBM and TPB theoretical models are applied to comprehensively explain the behavior of college students’ persuasion to their grandparents on COVID-19 vaccination, which may shed light on innovative and potentially feasible measures to improve vaccine uptake in older adults. Third, surveying older adults through college students not only simplifies personnel recruitment, but also avoids problems in the research process caused by dialect barriers and transportation inconveniences. Lastly, we will conduct a randomized controlled trial to determine the effect of college students’ persuasion on COVID-19 vaccination in older adults. Of note, China has more than 3000 colleges and more than 55 million college students [34]. With foreseeable potential cost effectiveness, this intervention will contribute to promote the formulation of working guidelines for the scale-up of COVID-19 vaccination among older adults in China and beyond.

This study faces potential challenges. First, the information of older adults reported by college students may not reflect their grandparents’ real situation. To mitigate this issue, participants who give the answer “unclear” to questions about older adults at Phase I will be excluded at Phase II. At Phase II participants will be asked to inquire their grandparents before completing the questionnaires. Second, the interval between Phase I and Phase II cannot be ignored. Grandparents’ COVID-19 vaccination status may change during this period. So we will double-check the actual vaccination status of grandparents to confirm participants’ eligibility. Third, the whole study procedure is based online, and the validity of the results will be affected [35, 36]. In our design, we target college students, who are highly educated and frequently accessed the Internet, to reduce the selection bias of the online survey [37]. Monetary incentives will be provided to reduce the risk of loss to follow-up [35]. The process of intervention will be followed by SMS reminders and quizzes to engage participants and improve the effect of web-based intervention. At the same time, through the logical verification of the three questionnaires and additional information about participants outside the questionnaire (IP address, response time, etc.), latent survey fraud can be partly prevented [38].

In summary, the knowledge gained from this study may be used to develop a grandchild-mediated strategy to promote COVID-19 vaccination in older adults, which may enrich vaccine promotion strategies for older adults in China and beyond.

## Supplementary Information

**Additional file 1.**

**Additional file 2.**

## Data Availability

The data collected in this study will not be publicly available. However, the corresponding author can be contacted for de-identified data on reasonable request.

## Abbreviations

COVID-19: Coronavirus disease 2019
KAP: Knowledge, attitude and practice
HBM: Health Belief Model
TPB: Theory of Planned Behavior
SMS: Short message service
