# Supplementary material for: College students’ influence on COVID-19 vaccination uptake among seniors in China: a protocol of combined cross-sectional and experimental study
Source: BMC Public Health. 2023 Jul 10;23:1322. doi: 10.1186/s12889-023-16209-2 (PMC10334528; doi:10.1186/s12889-023-16209-2)
Supplement: Supplementary file 1 — Additional file 1. [file 12889_2023_16209_MOESM1_ESM.docx]

**Items** **for** **Likert’s** **scales** **of** **HBM** **and** **TPB** **models**

| **Modules** | **Components** | **Items** | **Questionnaire** **code(s)** |
| --- | --- | --- | --- |
| HBM | Perceived susceptibility | If I don’t persuade, the risk of infection will be higher for my grandparents who have not received COVID-19 vaccine. | A |
|  |  | If I don’t persuade, the risk of severe or death will be higher for my grandparents who have not received COVID-19 vaccine. |  |
|  | Perceived severity | Even if my grandparents are infected with COVID-19, I don’t think it will cause serious health damage to them. |  |
|  |  | Even if my grandparents are infected with COVID-19, I believe there is a high probability of recovery. |  |
|  |  | Even if my grandparents are infected with COVID-19, I don't think it will cause too much family burden. |  |
|  | Perceived benefits | I believe COVID-19 vaccine can effectively prevent my grandparents from infection of COVID-19. |  |
|  |  | I believe COVID-19 vaccine can effectively prevent my grandparents from severe or death if infected with COVID-19. |  |
|  | Perceived barriers | Persuading grandparents to receive COVID-19 vaccine requires a lot of time to communicate. |  |
|  | Cues to action | Only with professionals’ guide in health education will I persuade my grandparents to receive COVID-19 vaccine. |  |
|  |  | Only when I have friends infected with COVID-19 will I persuade my grandparents to receive COVID-19 vaccine. |  |
|  |  | Only when the government promotes COVID-19 vaccination will I persuade my grandparents to receive COVID-19 vaccine. |  |
| TPB | Attitude | Persuading my grandparents to receive COVID-19 vaccine is a tedious process that requires time and effort. | A |
|  | Subjective norms | Persuading my grandparents to receive COVID-19 vaccine is a sign of filial piety. |  |
|  |  | Persuading my grandparents to receive COVID-19 vaccine is a sign of shouldering social responsibility. |  |
|  |  | Most of my friends will also persuade their grandparents to receive COVID-19 vaccine. |  |
|  | PBC* | I believe I can successfully persuade my grandparents to receive COVID-19 vaccine. |  |

Notes: HBM and TPB models are applied to explain college students’ willingness to persuade their grandparents to get COVID-19 vaccination. * PBC refers to perception of behavioral control.
