# Supplementary material for: College students’ influence on COVID-19 vaccination uptake among seniors in China: a protocol of combined cross-sectional and experimental study
Source: BMC Public Health. 2023 Jul 10;23:1322. doi: 10.1186/s12889-023-16209-2 (PMC10334528; doi:10.1186/s12889-023-16209-2)
Supplement: Supplementary file 2 — Additional file 2. [file 12889_2023_16209_MOESM2_ESM.docx]

**Items** **for** **Likert’s** **scales** **other** **than** **HBM** **and** **TPB** **models**

| **Modules** | **Items** | **Questionnaire** **code(s)** |
| --- | --- | --- |
| College students’ knowledge about COVID-19 vaccination in older adults | COVID-19 vaccination can significantly reduce the incidence, hospitalization rate and mortality of COVID-19 in older adults.  There is no maximum age limit for older adults to receive COVID-19 vaccine.  Older adults who always stay at home or rarely go out also need to receive COVID-19 vaccine.  Older adults without vaccination, especially those with chronic diseases, are more likely to develop severe COVID-19.  Older adults with chronic diseases and in a stable period can also receive COVID-19 vaccine.  It is necessary for older adults to receive a COVID-19 booster dose.  The efficacy of and duration of protection from COVID-19 vaccination among older adults is much lower than that among young people. | A |
| Grandparents’ attitude towards COVID-19 booster dose | It is beneficial to receive a COVID-19 booster dose.  It is harmful to receive a COVID-19 booster dose.  COVID-19 booster vaccination is essential to stop SARS-CoV-2 transmission and infection among older adults. | B&C |
| Grandparents’ intention to receive a COVID-19 booster dose | If I have passed the recommended interval between primary vaccination series and booster vaccination (6 months), I will receive a COVID-19 booster dose.  If without contraindication, I will receive a COVID-19 booster dose. | B&C |
